# Procedural Safety and Device Performance of the Portico™ Valve from Experienced TAVI Centers: 30-Day Outcomes in the Multicenter CONFIDENCE Registry

**DOI:** 10.3390/jcm11164839

**Published:** 2022-08-18

**Authors:** Helge Mollmann, Axel Linke, Luis Nombela-Franco, Martin Sluka, Juan Francisco Oteo Dominguez, Matteo Montorfano, Won-Keun Kim, Martin Arnold, Mariuca Vasa-Nicotera, Lenard Conradi, Anthony Camuglia, Francesco Bedogni, Ganesh Manoharan

**Affiliations:** 1 Department of Cardiology, St. Johannes Hospital, 44137 Dortmund, Germany; 2 Klinik für Innere Medizin/Kardiologie, Universitätsklinik Technische Universität Dresden, Herzzentrum Dresden Fetscherstraße 76, 01307 Dresden, Germany; 3 Cardiovascular Institute, Hospital Clinico San Carlos, Instituto de Investigación Sanitaria Hospital Clínico San Carlos (IdISSC), 28040 Madrid, Spain; 4 Department of Medicine-Cardiology, University Hospital Olomouc, 779 00 Olomouc, Czech Republic; 5 Interventional Cardiology Unit, Hospital Universitario Puerta de Hierro Hospital, 28222 Madrid, Spain; 6 Interventional Cardiology Unit, IRCCS Ospedale San Raffaele, 20132 Milan, Italy; 7 Kerckhoff Heart and Thorax Centre, 61231 Bad Nauheim, Germany; 8 Department of Cardiology, Friedrich Alexander Universität Erlangen-Nuremberg, 91054 Erlangen, Germany; 9 Klinikum der Johann Wolfgang Goethe Universitaet Frankfurt, 60596 Frankfurt, Germany; 10 Department of Cardiovascular Surgery, University Heart and Vascular Center, 20251 Hamburg, Germany; 11 Department of Cardiology, University of Queensland, Brisbane, QLD 4072, Australia; 12 Department of Cardiology, The Wesley Hospital, Brisbane, QLD 4066, Australia; 13 Department of Cardiology, IRCCS Policlinico San Donato, 20097 Milan, Italy; 14 Department of Cardiology, Royal Victoria Hospital, Belfast BT12 6BA, UK

**Keywords:** transcatheter aortic valve implantation, transcatheter aortic valve replacement, Portico, self-expanding, aortic stenosis

## Abstract

A total of 1001 subjects (82.0 years, 62.5% female, 63.7% NYHA III/IV at baseline) with severe aortic stenosis at high surgical risk were enrolled in the prospective CONFIDENCE registry and treated with a Portico™ transcatheter heart valve (THV) using either a first-generation delivery system (DS) or the FlexNav™ DS. The objective of this registry is to characterize the procedural safety and device performance of the Portico™ THV at 30 days. The study collected ‘standard-of-care’ clinical and device performance data, with adverse events adjudicated by an independent clinical event committee according to the Valve Academic Research Consortium-2 criteria. The implantation of a single Portico™ THV was successful in 97.5% of subjects. The 30-day all-cause mortality, cardiovascular mortality, and disabling stroke rates were 2.6%, 2.1%, and 1.8%, respectively. A new pacemaker was implanted in 19.0% of subjects at 30 days. At 30 days, the effective orifice area and mean gradient values were 1.82 cm^2^ and 7.1 mmHg, respectively. The 30-day rate of moderate paravalvular leak (PVL) was 2.1%, with no occurrence of severe PVL. The Portico™ THV demonstrated improved hemodynamic performance and low rates of safety events at 30 days in a large cohort of subjects implanted with the Portico™ THV with either the first-generation DS or FlexNav™ DS.

## 1. Introduction

Surgical aortic valve replacement (SAVR) was historically the primary treatment option for patients with severe, symptomatic aortic stenosis (AS). Various clinical trials and large-scale registries have compared SAVR to transcatheter aortic valve implantation (TAVI), showing a non-inferiority of TAVI vs. SAVR, first in patients at high and extreme surgical risk and later in those at intermediate and low surgical risk [1]. These findings have led to respective recommendations in the current guidelines for valvular heart disease, which describe TAVI as a safe and effective treatment for patients with severe, symptomatic AS [2,3].

The Portico™ valve (Abbott Structural Heart, Minneapolis, MN, USA) is a self-expanding transcatheter heart valve (THV) that first received the CE Mark in 2012 and received FDA approval with the FlexNav™ (Abbott Structural Heart, Minneapolis, MN, USA) Delivery System in 2021. As a condition of the CE Mark, the PORTICO I study was initiated to assess the procedural and 30-day outcomes and includes a follow-up of five years. The initial 30-day and 1-year outcomes from the PORTICO I study were previously reported [4,5]. PORTICO I represented early user experience of the Portico™ THV System. The CONFIDENCE registry was initiated to evaluate safety and performance of the Portico THV System at experienced Portico™ sites in a large cohort of high-risk or inoperable patients using standard clinical practice.

## 2. Materials and Methods

The **CON**trolled delivery **F**or **I**mprove**D** outcom**E**s with cli**N**i**C**al **E**vidence (CONFIDENCE) registry is a prospective, multicenter, single-arm, observational clinical investigation (ClinicalTrials.gov: NCT03752866). The objective of this registry is to characterize the procedural safety and device performance of experienced TAVI centers that commercially use Portico™ THVs to treat patients with severe symptomatic aortic stenosis at high or greater surgical risk. This clinical investigation includes 27 sites in 8 countries across Europe and 1 site in Australia. All the patients assessed for a commercial Portico™ THV implant at participating implantation centers were considered for inclusion in the registry. Experienced implanters, defined as those that have completed the commercial Portico™ implantation training program and performed at least 20 Portico™ THV implantations within the last 12 months, were invited to participate in the study. Consistent with other published post-market TAVI registries, the study collected ‘standard-of-care’ clinical and device performance data from experienced, high-volume TAVI implantation centers to ensure consistency with other published post-market TAVI registries.

Surgical risk assessment was performed per the institutional standard of care. The surgical risk was determined using risk calculators (STS PROM or EuroSCORE I/II), as well as by the local Heart Team who took additional factors into consideration, such as frailties and comorbidities not captured by the surgical risk calculators.

Patients that were of legal age in the host country, had severe symptomatic aortic stenosis, and an annulus range within the Portico™ THV sizing recommendations, were eligible for participation. This registry has broad inclusion criteria and minimal exclusion criteria to ensure that results are generalizable. The study’s inclusion and exclusion criteria are shown in Supplementary Table S1. All subjects who met all the inclusion criteria, did not meet any exclusion criteria, gave written informed consent, and had an attempted implant (defined as the point at which the delivery system (DS) enters the subject’s vasculature) were considered enrolled in the registry.

The study sites functioned in compliance with the Declaration of Helsinki, and approvals from ethics committees and local authorities were obtained. All patients provided written informed consent prior to participation. Abbott sponsored the study.

The Portico™ THV is a fully repositionable, self-expanding intra-annular valve within a nitinol frame. The valve cuff is made from porcine pericardium and is sutured to the stent frame. Four sizes of Portico™ THVs are available (23, 25, 27, and 29 mm) that cover native aortic annulus diameters of 19–27 mm and all were used in the CONFIDENCE registry.

The first cohort of subjects (*n* = 501) was implanted using the first-generation Portico™ DS (Abbott Structural Heart, Minneapolis, MN, USA), which requires a separate introducer sheath. The Portico™ THV was approved for use in access vessels ≥6 mm using an 18 Fr sheath (23 or 25 mm valve) or a 19 Fr sheath in vessels ≥6.5 mm (27 or 29 mm valve). Following the CE Mark of the next-generation FlexNav™ DS, a second cohort of subjects (*n* = 500) was implanted with the Portico™ THV. The FlexNav™ DS includes a hydrophilic-coated integrated sheath and stability layer to facilitate the gradual, controlled deployment of the Portico™ THV. The minimum access vessel size is reduced with the integrated sheath feature of the FlexNav™ DS to ≥5 mm for the smaller valve sizes (14 Fr equivalent integrated sheath diameter) and ≥5.5 mm for the larger valve sizes (15 Fr equivalent integrated sheath diameter).

Both the Portico™ DS and FlexNav™ DS allow for the repositioning of the Portico™ THV. The position of a partially deployed valve can be evaluated, and if needed, the valve can be resheathed and redeployed, provided the valve has not been fully deployed (beyond 80%) from the DS. The partially deployed valve may be resheathed up to two times at the implantation site.

Subjects underwent prospective enrollment with informed consent and baseline data collection (up to a maximum of 180 days prior to the Portico™ THV implantation procedure) prior to receiving their Portico™ THV. Pre-procedural multislice computer tomography (CT) was used (or echocardiography in some instances) to select the appropriate valve size (23, 25, 27, and 29 mm) for native aortic annulus diameters between 19 and 27 mm as per the Instructions For Use. The implantation procedure was conducted per the standard protocol established at each center. After the procedure, subjects underwent a pre-discharge visit at the time of hospital discharge or within seven days of the index procedure, whichever occurred first. Subjects returned to the participating institution for a 30-day follow-up visit, followed by a 12-month vital status/survival status check. 

Descriptive endpoints are reported for this registry using summary statistics. These endpoints include adverse event rates at 30 days from the index procedure (e.g., all-cause and cardiovascular mortality, myocardial infarction, stroke, bleeding, acute kidney injury, vascular complications), delivery profile characteristics, implant success, the echocardiographic assessment of valve performance at 30 days, and clinical improvement metrics at 30 days. An independent clinical event committee (CEC; Cardiovascular Research Foundation, New York, NY, USA) adjudicated all safety endpoints according to the VARC-2 guidelines [6]. Implant success was defined as the absence of procedural mortality and correct positioning of a single Portico™ THV into the proper anatomical location. 

Thirty-day echocardiograms were evaluated by an independent echocardiographic core laboratory (MedStar Health Research Institute, Washington, DC, USA). Paravalvular leak (PVL) was classified into four classes (none/trace, mild, moderate, and severe) according to the VARC-2 guidelines [6].

Continuous variables were summarized using the mean ± standard deviation. Categorical variables were summarized using frequencies and percentages. Paired Student’s *t*-tests (echocardiographic data) and the Wilcoxon signed-rank test (NYHA functional class) were used to compare outcomes at 30 days relative to the baseline. Evaluation of all adverse events was based on the CEC-adjudicated outcomes. The analysis population for the hemodynamic valve performance included only patients with a Portico™ THV implanted (i.e., a functioning Portico™ THV). A functioning Portico™ THV is defined as a Portico™ THV that is successfully deployed and functioning in the annulus, including those where more than one Portico™ THV is implanted in the annulus.

## 3. Results

### 3.1. Patients

**Figure 1:**
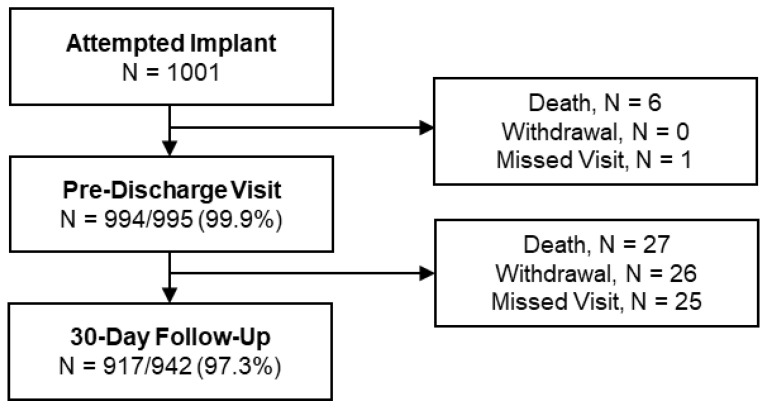
Subject disposition through 30 days after transcatheter aortic valve implantation. A total of 917 30-day visits were completed for 942 subjects eligible for follow-up.

Between October 2018 and July 2021, implantation with a Portico™ transcatheter heart valve (THV) was attempted in 1001 subjects (Figure 1). The first group of subjects was implanted using the first-generation Portico™ delivery system (DS) between October 2018 and January 2020 (i.e., the Portico™ DS cohort). The second group of subjects was implanted using the FlexNav™ DS between March 2020 and July 2021 (i.e., the FlexNav™ DS cohort). Follow-up compliance was 97.3% at the 30-day visit. The mean age was 82.0 ± 5.3 years, 62.5% were female, mean STS score was 4.2%, and 63.7% were in the NYHA class III/IV (Table 1). At least one frailty factor contributed to the estimation of surgical risk in 45.4% of the subjects. Hypertension was present in 87.0% of the subjects, coronary artery disease in 55.1%, cardiac arrhythmia in 48.4%, and diabetes in 35.8%. Prior percutaneous coronary intervention (PCI) and coronary artery bypass graft (CABG) occurred in 31.6% and 8.1% of the subjects, respectively. Of the 1001 subjects who underwent an implant with a Portico™ THV, 4 subjects (0.4%) had a prior surgical bioprosthesis (Mitroflow™, Sorin Group Inc., Milan, Italy). 

### 3.2. Procedural Characteristics

Table 2 provides procedural characteristics. Of the 1001 subjects, 976 (97.5%) were successfully implanted with a single Portico™ THV. Nineteen (19) subjects (1.9%) received a second valve (THV-in-THV), 7 of whom received a non-study valve. Three subjects (0.3%) had no Portico™ THV implanted; one had a non-study valve implanted due to difficult anatomy (i.e., horizontal aorta), in one subject the implanter had difficulty with the deployment of the Portico™ THV and the subject was implanted with a non-study valve, and in one subject the implanter attempted a Portico™ THV implant on two separate occasions but was unsuccessful on both occasions and ultimately implanted a non-study valve. Two subjects (0.2%) required conversion to surgical AVR; one due to dilatation of a non-study valve leading to annular rupture, and the other due to cardiogenic shock requiring an aortic valve and a mitral valve due to severe mitral regurgitation. Lastly, one subject died during the procedure (0.1%) due to an annular rupture believed to be caused by pre-implantation balloon valvuloplasty. Subjects with a non-study valve were followed for 30 days and exited the study following the resolution of any adverse events. Importantly, no procedural mortality or conversion to SAVR occurred in the FlexNav DS cohort.

Transfemoral access was obtained in the majority of subjects (98.7%), with the remaining subjects (1.3%) implanted via subclavian or axillary access. The average access vessel diameter was smaller in the FlexNav™ DS cohort than in the Portico™ DS cohort. An introducer sheath was used in 93.6% of the cases implanted with the Portico™ DS, compared with only 24.0% of the cases with the FlexNav™ DS. Pre-dilatation and resheathing were performed in 86.9% and 37.8% of the cases, respectively. The 27 mm valve size was used most often (35.7%), with the smaller valve sizes (23 and 25 mm) implanted more frequently in the FlexNav™ DS cohort. Post-dilatation occurred in 37.6% of the cases, with no significant differences between the cohorts. The average total procedure time from the first incision to closure was 69.0 min. 

### 3.3. VARC-2 Endpoints 

The 30-day descriptive endpoints are presented in Table 3. The all-cause mortality rate at 30 days was 3.2% in the Portico™ DS cohort and 2.0% in the FlexNav™ DS cohort, which included two (0.4%, 2/500) deaths related to COVID-19. Cardiovascular death occurred in 2.1% of subjects. Disabling stroke was observed in 18 subjects (1.8%), and stage 3 acute kidney injury occurred in 14 subjects (1.4%). Life-threatening bleeding occurred in 34 subjects (3.4%). Major vascular complications occurred in 73 subjects (7.3%), with the majority associated with access site complications. The access site complications of the TAVI delivery system across both cohorts (Portico™ DS cohort: 20, FlexNav™ DS cohort: 28) included closure device failure (*n* = 11), hematoma (*n* = 10), bleeding (*n* = 9), femoral artery injury (*n* = 6), pseudoaneurysm (*n* = 5), and singular events categorized as other (*n* = 7). A majority of the closure device failures (*n* = 8) occurred in the FlexNav™ DS cohort. A permanent pacemaker (PPI) was implanted in 171 subjects, representing 19.0% of the subjects with no prior pacemaker at baseline. There was no significant difference in new PPIs between the cohorts.

**Figure 2:**
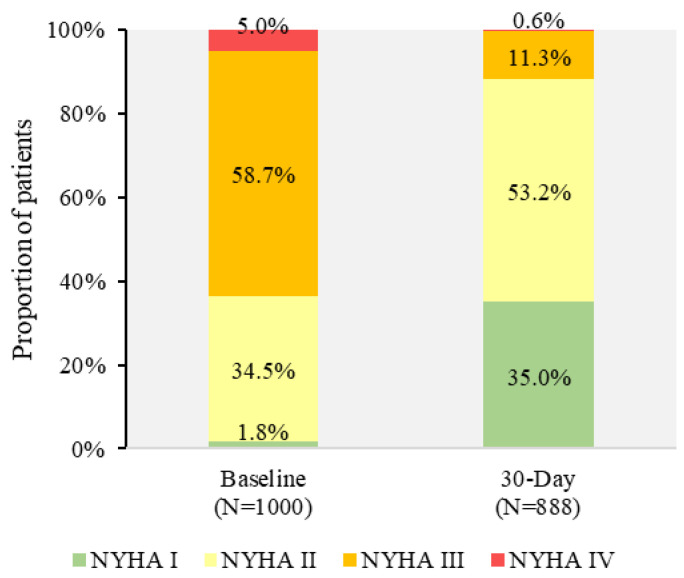
New York Heart Association functional class groups at 30 days (unpaired analysis).

Annular rupture occurred in three subjects (0.3%) caused by pre-dilatation of the balloon prior to Portico™ THV implantation in one subject (see the procedural mortality above), balloon expansion of a non-study valve leading to SAVR and ultimately death 10 days post-implantation in one subject, and perforation of the proximal ascending aorta by the stent frame in one subject. Five subjects (0.5%) experienced coronary obstruction; two due to a failing surgical bioprosthesis (Mitroflow™) and received a Portico™ THV (i.e., valve-in-valve), which required stenting; two subjects required PCI, and one subject had their Portico™ THV snared into the ascending aorta due to right coronary artery blockage and a non-study valve implanted in the annulus. Two subjects (0.2%, 2/1001) required a second valve within 30 days due to PVL; one subject received a second Portico™ THV and the other received a non-study valve. A majority of the subjects (88.2%) were in NYHA functional class I or II at 30 days (Figure 2). 

### 3.4. Hemodynamics

**Figure 3:**
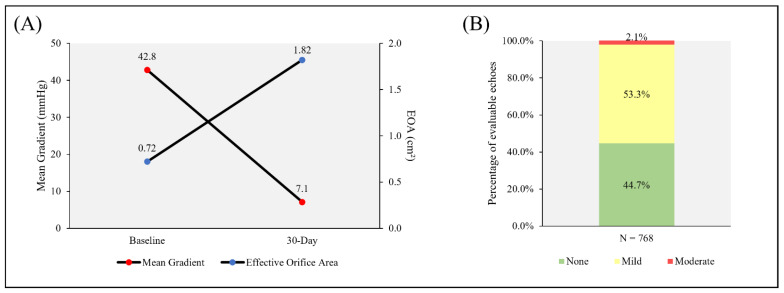
Valve hemodynamics: (**A**) effective orifice area (EOA) and mean gradient at baseline and 30 days; (**B**) severity of paravalvular leak at 30 days.

The effective orifice area (EOA) increased from 0.72 ± 0.18 cm^2^ at baseline to 1.82 ± 0.49 cm^2^ at 30 days (Figure 3A). The mean gradient improved from 42.8 ± 14.7 mmHg at baseline to 7.1 ± 3.7 mmHg at 30 days. PVL was mild or less in 97.9% of the subjects (Figure 3B). Moderate PVL was present in 2.1% of the subjects, with no cases of severe PVL.

## 4. Discussion

The CONFIDENCE registry is a large-scale prospective study of 1001 subjects with severe aortic stenosis who were treated with a Portico™ transcatheter heart valve (THV). The main findings of this real-world registry are: (1) high procedural success rate, (2) low 30-day mortality rate, and (3) favorable hemodynamic outcomes.

Subjects enrolled in the CONFIDENCE registry reflect the common TAVI population, with an average age of 82 years. Comorbidities such as prior cardiac arrhythmias, previous cardiovascular procedures, coronary artery disease, hypertension, and chronic kidney disease are consistent with high risk patient characteristics. 

The CONFIDENCE registry was divided into two cohorts: The first 501 subjects were implanted with a Portico™ THV valve via the first-generation Portico™ delivery system (DS), while the second 500 subjects were implanted using the FlexNav™ DS. The implantation of Portico™ THVs with either the Portico™ DS or FlexNav™ DS has previously been shown to be safe and effective in previous cohorts of high (or greater)-risk patients at 30 days [7,8,9]. 

The procedural characteristics were generally similar between the two cohorts, with the exception of the use of anesthesia, vessel diameter, and use of a separate introducer sheath. Given the low profile of the FlexNav™ DS and the ability to access vessels with diameters as low as 5 mm, the average vessel diameter was smaller in the second cohort implanted using the FlexNav™ DS compared with the first cohort implanted with the first-generation DS (7.09 mm vs. 7.42 mm, *p* = 0.0002). Since the FlexNav™ DS includes a hydrophilic-coated integrated sheath, subjects in this cohort were less likely to need a separate introducer sheath compared with those implanted using the first-generation DS (24.0% vs. 93.6%, *p* < 0.0001). Introducer sheaths were used in circumstances where the insertion of the FlexNav™ DS alone may have been difficult due to challenging anatomy or tortuosity.

The high procedural success rate (97.5%) is comparable to that of commercially available THVs [7,10,11,12]. The rate of severe complications was low. Root-cause analysis found that two of the three annular rupture events were caused by balloon dilatation. Additionally, two cases of coronary occlusion occurred after a valve-in-valve treatment of a failing surgical bioprosthesis; the higher risk of coronary occlusion in this specific procedure has been previously described [13]. 

The 30-day all-cause mortality rate of 2.6%, which includes two patients (0.2%) who died from COVID-19 in the FlexNav™ DS cohort, was low and represented a 2% lower 30-day mortality rate relative to that based on the surgical risk scores. This rate is similar to the rates observed (1.4–6.3%) in other registry studies of similarly high or greater risk patients [10,11,12,14,15]. In addition, the rate of all-cause mortality in the CONFIDENCE registry is slightly lower than the rates observed in previously published Portico™ THV studies (2.7–3.6%) [4,7]. 

The rate of major vascular complications at 30 days was 7.3%, with no statistical difference between the two cohorts (6.4% vs. 8.2%, *p* = 0.2311), and a majority of events occurring at the TAVI delivery system access site. Importantly, the CEC adjudicated only 1 of the 28 access site complications of the TAVI delivery system in the FlexNav™ DS cohort as possibly related to the delivery system; all others were not related. In comparison, 8 of the 20 site access complications of the TAVI delivery system in the first-generation Portico™ DS cohort were related to or possibly related to the delivery system. Although the FlexNav™ DS cohort experienced a higher major vascular complication rate than the Portico™ DS cohort, the rate of delivery-system-related major vascular complications was lower with the FlexNav™ delivery system.

The rate of naïve PPI in this registry is comparable to that of most commercially available self-expandable THVs in high or greater risk patients. The observed rate of 19.0% in the CONFIDENCE registry is in line with naïve PPI rates reported in the PORTICO I (18.7%, Portico), FORWARD (19.3%, Evolut™ R, Medtronic, Minneapolis, MN, USA), and FORWARD PRO (20.7%, Evolut™ PRO, Medtronic, Minneapolis, MN, USA) registries at 30 days [4,10,14]. Pre-existing right bundle branch block and annulus size have been identified as independent predictors for pacemaker need after Portico™ implantation [16]. However, the new PPI rate observed in the CONFIDENCE registry is higher than the rate of the Acurate neo™ THV (Boston Scientific, Marlborough, MA, USA) in the SAVI TF registry (8.3%) at 30 days [12]. While the PPI rate in the CONFIDENCE registry is in line with most other self-expandable THVs, it is higher than the rates reported in balloon-expandable THV registry studies (9.5–12.0%) [11,15]. Continuing to improve the implantation technique to achieve a higher valve implantation depth and reducing valve manipulations prior to final deployment may help reduce the overall rate of PPI. Further investigation is needed to accomplish this goal. 

Hemodynamic performance at 30 days showed clinically relevant improvement in the effective orifice area and mean gradient from baseline. The rate of moderate PVL at 30 days was 2.1%, with no instances of severe PVL. This rate is similar to the rate of PVL at discharge for Evolut™ R THVs in the FORWARD study (2.0% moderate, 0.1% severe PVL), and lower than the rate for ACURATE neo™ THVs in the SAVI TF study (4.1% moderate) [10,12]. The low rate of moderate PVL in the CONFIDENCE registry is noteworthy, given that Portico™ THVs do not have a dedicated outer sealing skirt like the commercially available Sapien™ 3 (Edwards Lifesciences, Irvine, CA, USA) and Evolut™ PRO THVs. For reference, the Sapien™ 3 THV had 3.0% moderate and 0.1% severe PVL at 30 days, and the Evolut™ PRO THV had a combined moderate-and-severe PVL rate of 1.6% at discharge [11,14].

The Portico valve was designed with large open cells as a strategy to mitigate PVL with the single tissue cuff design. The large open stent cells in the annulus region maximize cuff sealing tissue and lowers the probability of a stent strut being directly opposed against bulky calcium nodules as compared to TAVI stents with higher stent cell density like Evolut™ and Sapien™ TAVI systems. The ability to maximize tissue contact with bulky calcium and minimize stent struts that create blood channels explains why Portico has low PVL occurrence rates despite not having an outer PVL cuff.

The Portico™ THV has an intra-annular leaflet design that, together with the wide cells of the valve stent, allows for easy access to the coronary ostia after valve implantation [17,18]. The intra-annular leaflet design was believed to have an inherent risk of higher residual gradients, as presented in a recent echo Doppler comparative study of both self-expanding and balloon-expandable THVs [19]. The average mean gradient of 7.1 mmHg at 30 days for Portico™ THVs is lower than those observed for the intra-annular Sapien™ XT (10.2 mmHg) and Sapien™ 3 (11.9 mmHg) THVs. The average mean gradient in the CONFIDENCE registry is more in line with supra-annular THVs, such as the Evolut™ R (8.5 mmHg) and Evolut™ PRO (7.9 mmHg) THVs [10,11,14,15]. 

Implantation of the Portico™ THV was associated with functional improvement at 30 days. The reduction in NYHA classes III/IV from 63.5% at baseline to 11.9% at 30 days is a markedly positive improvement. This outcome is in line with the clinical improvement observed at 30 days in both self-expanding (8.0–12.0%) [10,12,14] and balloon-expandable (10.0–10.4%) [11,15] THVs.

## 5. Conclusions

The CONFIDENCE registry demonstrates favorable short-term clinical and echocardiographic outcomes for subjects with severe aortic stenosis treated with Portico™ THVs. The FlexNav™ delivery system allowed for the treatment of subjects with smaller access site vessels, resulting in an improved implantation depth and fewer access-site vascular complications related to the delivery system. 

The limitations of the CONFIDENCE registry are those inherent to non-interventional registries. The strengths of this registry are the large number of patients, the high subject follow-up at 30 days (97.3%), the evaluation of echocardiographic data by an independent core lab, and the adjudication of adverse events by an independent clinical events committee

## Figures and Tables

**Table 1:** Demographics and clinical characteristics.

Characteristic	Portico™ DS ^1^ % (*n*) of Subjects (*n* = 501)	FlexNav™ DS % (*n*) of Subjects (*n* = 500)	Total (*n* = 1001)
Age (Years)	81.7 ± 5.4	82.3 ± 5.3	82.0 ± 5.3
Gender (Female)	63.7%	61.4%	62.5%
NYHA Class			
I	2.8%	0.8%	1.8%
II	31.9%	37.0%	34.5%
III	58.9%	58.6%	58.7%
IV	6.4%	3.6%	5.0%
EuroSCORE I (%)	16.4 ± 11.1	14.9 ± 10.3	15.7 ± 10.8
EuroSCORE II (%)	4.8 ± 3.8	4.7 ± 4.3	4.8 ± 4.1
STS Mortality Risk Score (%)	4.2 ± 2.9	4.2 ± 2.7	4.2 ± 2.8
Number of frailty factors contributing to the subject’s surgical risk score			
1	23.5%	19.3%	21.1%
2	14.7%	11.8%	13.1%
3	9.7%	10.7%	10.2%
4	0.9%	1.2%	1.0%
Cardiac arrhythmia	49.1%	47.6%	48.4%
Carotid artery disease	12.6%	10.6%	11.6%
Chronic kidney disease	27.7%	26.0%	26.9%
Dialysis	2.9%	1.5%	2.2%
Chronic lung disease	19.4%	21.0%	20.2%
Coronary artery disease	57.9%	52.4%	55.1%
Diabetes	35.1%	36.4%	35.8%
Dyslipidemia	59.3%	64.4%	61.8%
Hematologic disorders	10.4%	10.8%	10.6%
Hypertension	87.8%	86.2%	87.0%
Liver disease or cirrhosis	3.4%	3.0%	3.2%
Mitral valve disease	61.7%	61.0%	61.3%
Myocardial infarction	13.6%	12.2%	12.9%
Peripheral artery disease	12.0%	11.8%	11.9%
Prior permanent pacemaker	9.4%	11.2%	10.3%
Prior CABG	7.4%	8.8%	8.1%
Prior PCI	31.7%	31.4%	31.6%
Prior stroke	10.6%	7.6%	9.1%
Prior TIA	4.4%	5.0%	4.7%
Mean aortic valve gradient (mmHg)	43.4 ± 14.5	42.2 ± 15.0	42.8 ± 14.7
Aortic valve area (cm^2^)	0.71 ± 0.2	0.72 ± 0.2	0.72 ± 0.18

^1^ First-generation Portico™ delivery system.

**Table 2:** Procedural characteristics.

Characteristic	Portico™ DS ^1^ % (*n*) of Subjects (*n* = 501)	FlexNav™ DS % (*n*) of Subjects (*n* = 500)	*p*-Value	Total % (*n*) of Subjects (*n* = 1001)
Portico™ valve implant success	97.4% (488)	97.6% (488)	0.8434	97.5% (976)
Procedural mortality	0.2% (1)	0.0% (0)	1.0000	0.1% (1)
Conversion to SAVR	0.4% (2)	0.0% (0)	0.4995	0.2% (2)
More than 1 valve implanted ^2^	1.8% (9)	2.0% (10)	0.8134	1.9% (19)
No Portico™ valve implanted	0.2% (1)	0.4% (2)	0.6242	0.3% (3)
Anesthesia			<0.0001	
General anesthesia	30.1% (151)	16.4% (82)		23.3% (233)
Conscious sedation	69.9% (350)	82.6% (413)		76.2% (763)
Access method			0.1639	
Transfemoral	98.2% (492)	99.2% (496)		98.7% (988)
Subclavian/axillary	1.8% (9)	0.8% (4)		1.3% (13)
Vessel diameter (mm)	7.42 ± 1.44	7.09 ± 1.36	0.0002	7.25 ± 1.41
Valve utilized				
23 mm	5.6% (28)	9.0% (45)	0.0380	7.3% (73)
25 mm	25.7% (129)	31.4% (157)	0.0478	28.6% (286)
27 mm	39.1% (196)	32.2% (161)	0.0223	35.7% (357)
29 mm	29.5% (148)	27.4% (137)	0.4530	28.5% (285)
Valve resheathed	34.3% (172)	41.2% (206)	0.0250	37.8% (378)
Implant too high	55.8% (96)	77.7% (160)		67.7% (256)
Implant too low	35.5% (61)	18.0% (37)		25.9% (98)
Coronary occlusion	0.0% (0)	0.0% (0)		0.0% (0)
Other	8.7% (15)	4.4% (9)		6.3% (24)
Introducer sheath used	93.6% (469)	24.0% (120)	<0.0001	58.8% (589)
18F	32.8% (154)	25.8% (31)		31.4% (185)
19F	65.7% (308)	65.0% (78)		65.5% (386)
20F	0.2% (1)	4.2% (5)		1.0% (6)
Other	1.3% (6)	5.0% (6)		2.0% (12)
Pre-balloon valvuloplasty	85.4% (428)	88.4% (442)	0.1635	86.9% (870)
Post-balloon valvuloplasty	37.7% (189)	37.4% (187)	0.9156	37.6% (376)
Concomitant procedures	8.4% (42)	4.8% (24)	0.0224	6.6% (66)
Non-coronary cusp (NCC) depth (mm)	5.1 ± 3.0	4.5 ± 3.0	0.0043	4.8 ± 3.0
Left coronary cusp (LCC) depth (mm)	5.8 ± 2.9	5.5 ± 2.9	0.0610	5.6 ± 2.9
Total procedure time (first incision to closure, min)	64.6 ± 38.0	73.4 ± 39.8	0.0004	69.0 ± 39.1

^1^ First-generation Portico™ delivery system; ^2^ includes more than one Portico™ THV or Portico™ THV and other commercial valve implanted.

**Table 3:** The 30-day outcomes.

Event Type	Portico™ DS % (*n*) of Subjects (*n* = 501)	FlexNav™ DS % (*n*) of Subjects (*n* = 500)	*p*-Value	Total: % (*n*) of Subjects (*n* = 1001)
All-cause mortality	3.2% (16)	2.0% (10) ^1^	0.6376	2.6% (26)
Cardiovascular mortality ^2^	3.0% (15)	1.2% (6)	0.1270	2.1% (21)
Non-cardiovascular mortality	0.2% (1)	0.8% (4)	0.1516	0.5% (5)
Myocardial infarction	0.4% (2)	0.2% (1)	1.0000	0.3% (3)
Acute kidney injury stage	1.4% (7)	0.8% (4)	0.3841	1.1% (11)
Bleeding events				
Life-threatening	3.2% (16)	3.6% (18)	0.6755	3.4% (34)
Major bleeding	5.2% (26)	6.6% (33)	0.3020	5.9% (59)
Minor bleeding	6.2% (31)	4.0% (20)	0.1903	5.1% (51)
Stroke				
Disabling	1.6% (8)	2.0% (10)	0.5989	1.8% (18)
Non-disabling	1.0% (5)	1.2% (6)	0.7320	1.1% (11)
Vascular complications	13.2% (66)	12.6% (63)	0.8250	12.9% (129)
Major vascular complications	6.4% (32)	8.2% (41)	0.2311	7.3% (73)
Access site complication	6.0% (30)	7.4% (37)	0.3249	6.7% (67)
TAVI Delivery System site	4.0% (20)	5.6% (28)	0.2054	4.8% (48)
Non-TAVI Delivery System site	2.0% (10)	1.8% (9)	0.8573	1.9% (19)
Non-access site complication	0.4% (2)	0.8% (4)	0.4466	0.6% (6)
Minor vascular complications	7.2% (36)	5.0% (25)	0.1108	6.1% (61)
Naïve permanent pacemaker insertion ^3^	19.2% (87)	18.9% (84)	0.9777	19.0% (171)
Annular rupture	0.4% (2)	0.2% (1)	1.0000	0.3% (3)
Coronary obstruction	0.4% (2)	0.6% (3)	0.6833	0.5% (5)

^1^ Includes two (0.2%) COVID-19 deaths adjudicated by the CEC as non-cardiovascular; ^2^ unknown mortality is classified as cardiovascular mortality; ^3^ among patients without a pacemaker at baseline.

## Data Availability

The data presented in this study are available on request from the corresponding author.

## Supplementary Materials

The following supporting information can be downloaded at: https://www.mdpi.com/article/10.3390/jcm11164839/s1, Table S1: Study inclusion and exclusion criteria.
