# Supplementary material for: Procedural Safety and Device Performance of the Portico™ Valve from Experienced TAVI Centers: 30-Day Outcomes in the Multicenter CONFIDENCE Registry
Source: J Clin Med. 2022 Aug 18;11(16):4839. doi: 10.3390/jcm11164839 (PMC9409954; doi:10.3390/jcm11164839)
Supplement: Supplementary file 1 [file jcm-11-04839-s001.zip › jcm-1794548-supplementary.pdf]

Procedural safety and device performance of the Portico™ valve from experienced TAVI centers: 30-day outcomes in the multicenter CONFIDENCE registry

Supplementary Materials

Table S1. Study Inclusion and Exclusion Criteria

Inclusion Criteria	
1.	Are ≥ 18 years of age or legal age in host country and have been identified as a candidate for a Portico™ THV implant
2.	Have provided written informed consent as approved by the Ethics Committee of the respective clinical center
Exclusion Criteria	
1.	Have sepsis, including active endocarditis
2.	Have any evidence of left ventricular or atrial thrombus
3.	Have vascular conditions that make insertion and endovascular access to the aortic valve improbable (e.g. caliber, stenosis, tortuosity, severe calcification)
4.	Have a non-calcified aortic annulus
5.	Have a congenital bicuspid or unicuspid leaflet configuration
6.	Are unable to tolerate antiplatelet/anticoagulant therapy
7.	Are pregnant at the time of signing informed consent
8.	Are currently participating in a drug or device study that may impact the registry (unless prior sponsor approval for co-enrollment is granted)
